# Vapor-fed bio-hybrid fuel cell

**DOI:** 10.1186/s13068-017-0755-7

**Published:** 2017-03-17

**Authors:** Marcus S. Benyamin, Justin P. Jahnke, David M. Mackie

**Affiliations:** Army Research Laboratory, 2800 Powder Mill Road, Adelphi, MD 20740 USA

**Keywords:** Waste management, Waste-to-energy, Bio-hybrid, Fuel cell, Ethanol, Vapor, Yeast, Fermentation, Biofuel

## Abstract

**Background:**

Concentration and purification of ethanol and other biofuels from fermentations are energy-intensive processes, with amplified costs at smaller scales. To circumvent the need for these processes, and to potentially reduce transportation costs as well, we have previously investigated bio-hybrid fuel cells (FCs), in which a fermentation and FC are closely coupled. However, long-term operation requires strictly preventing the fermentation and FC from harming each other. We introduce here the concept of the vapor-fed bio-hybrid FC as a means of continuously extracting power from ongoing fermentations at ambient conditions. By bubbling a carrier gas (N_2_) through a yeast fermentation and then through a direct ethanol FC, we protect the FC anode from the catalyst poisons in the fermentation (which are non-volatile), and also protect the yeast from harmful FC products (notably acetic acid) and from build-up of ethanol.

**Results:**

Since vapor-fed direct ethanol FCs at ambient conditions have never been systematically characterized (in contrast to vapor-fed direct methanol FCs), we first assess the effects on output power and conversion efficiency of ethanol concentration, vapor flow rate, and FC voltage. The results fit a continuous stirred-tank reactor model. Over a wide range of ethanol partial pressures (2–8 mmHg), power densities are comparable to those for liquid-fed direct ethanol FCs at the same temperature, with power densities >2 mW/cm^2^ obtained. We then demonstrate the continuous operation of a vapor-fed bio-hybrid FC with fermentation for 5 months, with no indication of performance degradation due to poisoning (of either the FC or the fermentation). It is further shown that the system is stable, recovering quickly from disturbances or from interruptions in maintenance.

**Conclusions:**

The vapor-fed bio-hybrid FC enables extraction of power from dilute bio-ethanol streams without costly concentration and purification steps. The concept should be scalable to both large and small operations and should be generalizable to other biofuels and waste-to-energy systems.

**Electronic supplementary material:**

The online version of this article (doi:10.1186/s13068-017-0755-7) contains supplementary material, which is available to authorized users.

## Background

Biofuels have become an increasingly important source of energy over the past several decades, and of these fuels, ethanol is the most advanced, with well-established production and distribution [1, 2]. It is already a major gasoline additive in many countries, and is a stand-alone transportation fuel in Brazil. Ethanol has an intermediate specific energy density of 30 MJ/kg, between that of gasoline (45 MJ/kg) and methanol (23 MJ/kg), and higher than the sugar from which it is derived (16 MJ/kg) [3–5]. It has the advantage that microbial fermentations can produce ethanol from a variety of feedstocks [6–11], more quickly and at higher concentrations than other biofuels [12–14]. The yeast *Saccharomyces cerevisiae* is particularly quick and efficient in producing ethanol from simple sugars. For example, Brazilian industrial ethanol production from cane sugar and molasses achieves concentrations of 8–11% (*v*/*v*) within a period of 6–11 h at 32–35 °C [15]. Currently used industrial strains of *S. cerevisiae* are also capable of producing high titers of ethanol, which reduces the energetic and economic costs of downstream processing, and further enhancements are possible through genetic manipulation [16].

There has been significant interest in using biofuels such as ethanol with fuel cells (FCs). In part this is because with combustion-driven power generation much of a biofuel’s inherent energy content is pre-spent in purification, organism recycling, and water removal [17]. Distillation alone can consume about a third of the total chemical energy in the biofuel [18, 19]. Pervaporation membranes are under development as a more efficient means of concentrating biofuels, particularly in dilute fuel streams [20]. Transportation costs also, both for biofuels and their pre-cursors, are significant (and often overlooked) [21, 22]. These parasitic energy losses make bio-ethanol a less viable alternative to fossil fuels for combustion, from both an environmental and economic standpoint. In contrast, FCs are able to operate efficiently using dilute fuel streams [23–25]. In particular, proton-exchange membrane FCs actually require water for the anode reaction and to keep the FC’s Nafion proton-exchange membrane (PEM) ionically conductive [26], so the most costly production step—water removal—can be skipped, at least in principle.

Further, since FCs are not limited by the Carnot cycle, in theory they can be extremely efficient [27]. PEM FCs using hydrogen as fuel have achieved efficiencies over 50% [28], and >70% efficiency has been demonstrated by solid oxide FCs with waste heat captured by a turbine [29, 30]. At ambient or slightly elevated temperature, methanol FCs have lower efficiencies, around 30%, due to higher reaction overpotential and methanol crossover [31]. Currently available direct ethanol FCs have low efficiencies near room temperature, mainly because their anode catalysts rarely break carbon–carbon bonds. But research is ongoing into improving metal catalysts [32, 33], developing enzymatic catalysts [34, 35], and improving membranes [32, 36, 37]. In the meantime, ambient-temperature direct ethanol FCs serve well as proxies for other FC technologies, while being readily available and easy to operate, over a range of scales.

Bio-hybrid FCs attempt to link the biotic and abiotic steps of fuel production and use as closely as possible. A microbial culture produces a biofuel, and the culture liquid (fermentate) is used directly in a FC to produce electrical power with minimal processing [38, 39]. Of course, this introduces complications, because the biotic and abiotic processes interact harmfully with each other. For instance, thiol and amine groups on proteins (present in rich growth media) will bind readily and irreversibly to deactivate noble metal catalysts, such as the platinum–ruthenium on direct ethanol FC anodes [40, 41]. In our experience, this deactivation takes only minutes, and is difficult to undo [39]. Also, excessive alkali metal cations (e.g., Na^+^, K^+^) that are present in many growth media block the H^+^ conduction channels in PEMs [42]. Prior work has demonstrated that spent yeast fermentations can be used in a direct ethanol FC without purification if the growth medium minimizes such components [39]. Further work has shown that the use of rich media (allowing for faster ethanol production) with a bio-hybrid direct ethanol FC is possible, provided that a reverse osmosis (RO) membrane is used to passively separate the anode from the fermentation [38]. However, over long time periods (1 week), catalyst poisoning decreases FC performance, presumably from amino acids that manage to get past the RO membrane [43]. In the opposite direction, there is also the problem that at near-ambient temperature the primary oxidation product of Pt–Ru direct ethanol FCs is acetic acid rather than carbon dioxide [44–47]. Acetic acid readily back-diffuses through an RO membrane and kills the yeast at one or two percent concentration [48]. Other solvent-using technologies and solvent-generating organisms are likely to have similar issues of fouling and product inhibition, respectively [49] (e.g., alcohols to aldehydes [50], carbon dioxide capture by ethanol and ammonia [51], *E. coli* genetically modified to produce ethanol [52], and ABE bacteria [53]). Thus, while there are significant energetic and economic benefits to combining the biotic and abiotic processes that together produce electrical power, some decoupling of fermentation and FC also seems desirable, and is perhaps unavoidable [54].

**Fig. 1:**
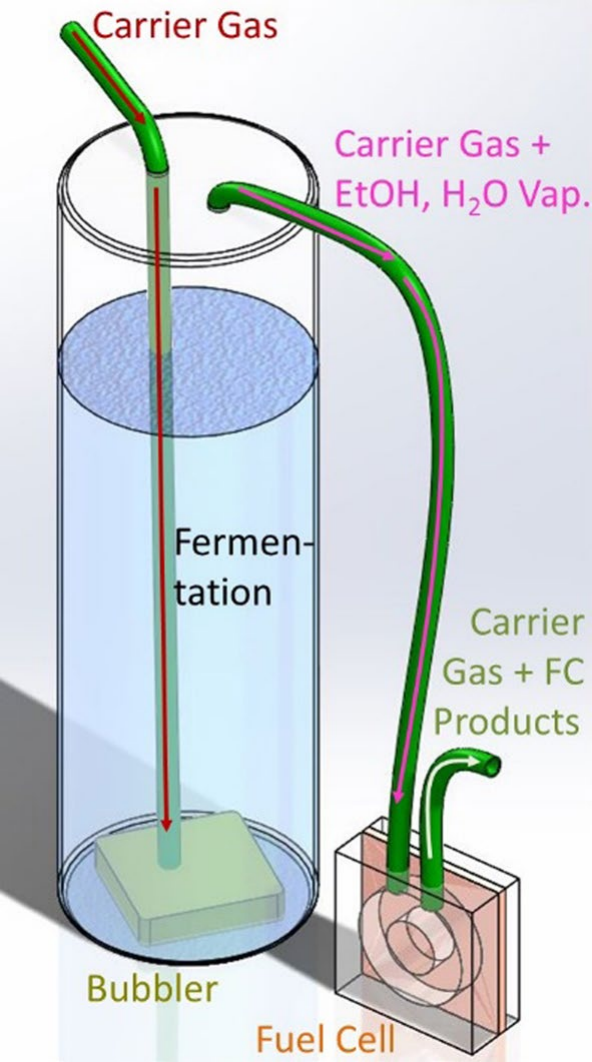
Schematic diagram of vapor-fed bio-hybrid fuel cell (FC). Carrier gas is pumped through a bubbler and into an ongoing fermentation. This removes ethanol and water as a vapor in the bubbles, leaving behind non-volatiles. The ethanol/water vapor is fed into a FC to produce electrical power. The exhaust may be processed further (not shown) to recover water, carrier gas, and unused fuel

An alternative method of delivering ethanol from the fermentation to the FC would be transferring ethanol via the vapor phase by the bubbling of an inert gas as shown in Fig. 1 (industrially known as “stripping”). Stripping and similar methods (e.g., pervaporation) have been investigated as methods for removing solvents from fermentations, often in combination with later distillation steps [19, 53]. They generally have lower energetic costs than distillation by itself, especially at low solvent concentrations [55]. (This has made them particularly relevant for acetone–butanol–ethanol fermentations where butanol concentrations of only a few weight percent kill the organisms [53].) These methods should strongly discriminate against non-volatile compounds present in the media. In particular, salts and polypeptides are known to be highly non-volatile [56]. Thus, while ethanol and water vapor are carried to the anode of the FC, harmful components should be left almost exclusively in the fermentation, preventing them from poisoning the FC catalyst. Moreover, there is the additional advantage that waste acetic acid from the FC should be washed out by the flowing gas, along with any excess ethanol or water vapor, protecting the FC and the fermenting organism(s) from acetic acid poisoning. In summary, this setup should allow a FC to be run directly off the fermentation without any additional processing. However, this requires ensuring several things: that the FC can operate at reasonable power densities on the concentrations of ethanol vapor that can be obtained in the gas stream; that the concentration of harmful components in the gas stream is low enough to minimize poisoning of the FC catalyst; and that the acetic acid is indeed kept from returning to the fermentation.

Interestingly, while direct ethanol FCs have been run on ethanol vapors at elevated temperatures [44], very little work has been done examining the behavior of vapor-fed direct ethanol FCs at room temperature [57], and no systematic study has been published. This is despite the fact that a great deal of work has been done on vapor phase operation of direct methanol FCs and has demonstrated benefits to running FCs on vapor feeds [58–61]. For example, in liquid phase operation, the methanol concentration must be kept low to reduce crossover and mixed potential effects but vapor phase operation can use more concentrated methanol, increasing the energy density of the carried fuel [62]. While fuel crossover is not such a major issue for direct ethanol FCs, one would expect benefits to near-ambient vapor phase operation of direct ethanol FCs, especially if they are coupled to a fermentation as a bio-hybrid FC.

Here we report the operation and performance characteristics of a novel ambient-temperature (25 °C) vapor-fed bio-hybrid FC. It is shown that the power and current densities obtained from vapor-fed operations are comparable to the values for liquid-fed operation, and that the FC can run near-optimally over a wide range of ethanol concentrations (2–8 mmHg, equivalent to saturated gas obtained from bubbling through 3–15% ethanol solutions). Having characterized the vapor-fed FC, its long-term potential is demonstrated by operating it for 5 months in combination with a yeast fermentation to supply the ethanol. This study shows that a vapor-fed FC has the potential to be operated in continuous mode over long periods of time, in contrast to previous work [63]. In addition, the ethanol production rate is increased, presumably by preventing ethanol from reaching concentrations that retard fermentation.

## Methods

The overall setup has three parts. (Compare Additional file 1: Figure S1, § Overall Setup.) In the first part, nitrogen is bubbled through a fermentation or an ethanol–water mixture. In the second part, the bubbled nitrogen carries ethanol and water vapor into a FC, which produces electrical power (carbon dioxide from fermentations would travel this same route, but is not expected to affect FC performance [64]). The optional third part is a cold trap, and is only for evaluating the output of the first or second part. The three parts are detailed in the sections below.

Water was deionized with a reverse osmosis membrane and passed through a Barnstead Nanopure water polisher. Nitrogen gas was house dry nitrogen originating in liquid nitrogen boil-off. Glucose and ethanol were from Sigma, >99.5% purity, and ethanol was ACS reagent grade. Yeast extract was of microbiology grade, from Sigma, and bacteriological peptones were from Fluka. All reagents were used as-received.

### Vapor delivery system

Ethanol in the vapor phase was obtained by bubbling (sparging) nitrogen through either ethanol–water mixtures or ongoing fermentations, using a glass-bonded air diffuser with a maximum pore size of 80 μm (Bubblemac, BB1030F). The height of the bubbling column was 15 cm. Preliminary experiments showed that this height was sufficient to saturate the nitrogen with ethanol and water vapor at even the highest flow rate (see Additional file 1: § Vapor–Liquid Equilibrium). The output gas mixture was sent either directly to the cold trap (for evaluation), or through the FC anode reservoir (to provide fuel for electrical power). The nitrogen flow rate was controlled with flow meters (Cole-Parmer, UX-32470 series) or mass flow controllers (Omega, FMA 5500 series), depending on the flow rate. The temperature of the bubbling apparatus was held just above room temperature (23–25 °C) to keep it stable, using a digital incubator (Boekel, 133000). For the given ratios in the ethanol–water mixture and a given temperature, the partial pressures of ethanol and water being carried by the nitrogen were predicted using Wilson’s equation [65]. Predictions were verified using the cold trap; see section “Cold trap” below.

### Fermentations

The *S. cerevisiae* VL3 wine yeast strain (Zymaflore, Bordeaux, France) was used in all experiments and was always grown in rich YPD media, containing 2% yeast extract, 2% bacteriological peptone, and dextrose (glucose). YPD media models many of the aspects of a sugar-rich food waste stream, with lipids and proteins of various sizes. It is known to rapidly poison FCs such as those used here [39, 48]. The initial glucose concentration was 6% (w/v). Glucose was added in increments of 7.5 g (6%) or 3.75 g (3%) equally to bubbled and control fermentations at regular intervals (usually twice per week). Starting liquid volume was 125 mL. Either 5 or 10 mL of water was also added weekly to the bubbled fermentation to replace the water lost in vapor. In the long-term bubbled fermentation, the glucose concentration was allowed to vary widely, between 0.5 and 6%, to test the stability of the system. Prior to using this fermentation with the vapor-fed FC, nitrogen was bubbled through it at a flow rate of 30 mL/min. After 24 h, the output gas mixture was connected to the other parts of the experiment. Control fermentations were prepared and maintained in the same manner, except without any nitrogen bubbling. The concentrations of fermentation components were determined using IR spectroscopy, just prior to additions of glucose and water.

### Fourier transform infrared (FTIR) spectroscopy

IR spectra were obtained with an Alpha FTIR spectrometer (Bruker Optics Inc.: Billerica, MA, USA) with a diamond attenuated total reflection attachment. To determine the composition of samples, the sample spectra were deconvolved using reference spectra and a least squares fitting method [66]. Typically, 48 scans were collected on a 50 μL sample and water backgrounds were collected prior to measurement. The methodology was tested extensively against known mixtures and compared to HPLC and was found to have an absolute error in the concentration percentage of 0.1% or less (for example, a concentration reported as 2.3% was 2.2–2.4%, at the 95% confidence level).

### Fuel cells

The FC components of the vapor-fed FC system were typical direct methanol FCs that were obtained from fuelcellstore.com (SKU 1071041, H-Tec Ind., GmbH, single plate methanol/air PEMFC that had 2.68 cm^2^ of active area, where active area is the measured area of the anode). These FCs were chosen to be consistent with our previous studies, in which we ran them in the conventional manner, as liquid-fed direct ethanol FCs [39, 48]. The membrane electrode assembly (MEA) has Pt–Ru/C as the anode catalyst, Pt/C as the cathode catalyst, and a Nafion^®^ 117 proton-exchange membrane. The acrylic housing for the MEA has a cylindrical reservoir (1.5 mL) on the anode side with two fill holes. When the FCs were run with vapor feeds, these two holes were used as the vapor inlet and outlet. Prior to use, the FCs were cleaned by soaking in 5% (*v*/*v*) sulfuric acid overnight, per manufacturer’s instructions. When in use, the FCs were kept in the same digital incubator as the ethanol–water mixture (or the fermentation), at 23‒25 °C.

### Electrical measurements

Electrochemical characterization of the vapor-fed FC system was done with an eight channel VMP3 potentiostat obtained from Biologic (Claix, France). The FC’s cathode was used as both a pseudo-reference and as the counter electrode. Performance of FCs was characterized with chronoamperometric (I-t) measurements poised at 200 mV, and with variable voltage measurements between 0 and 500 mV. In the former, 200 mV was chosen because previous experiments had shown that was near the optimal voltage for the FCs being used, when fueled with liquid ethanol/water in batch mode. In the latter, the vapor-fed FCs were held at 200 mV for 2 h to reach a steady state before the voltages were varied, and the current was recorded. The voltage was stepped and held for 20 min at each target voltage, then stepped and held at 200 mV in between target voltages. This ensured that the vapor-fed FC system could reach a steady state at each voltage. The voltages were stepped rather than continuously swept, because we were unsure of the response time of the vapor-fed FC system. Although the voltage is stepped discontinuously rather than swept, the data produced are similar to a linear sweep voltammogram (LSV). The power is calculated as the product of the voltage (potential, which is set by the electrochemical workstation) and the measured output current of the FC. All error bars shown on the electrochemical data represent the standard deviation for at least three independent runs. Where error bars are not shown, such as some of the voltage sweeps and the long-term bio-hybrid FC, only a single run was done; the uncertainty is expected to be similar to data where multiple runs were done.

Conversion values were obtained by integrating current measurements, and the plotted results are based on the assumptions that Faradaic losses are negligible, and that all ethanol is converted to acetic acid (with 4 electrons produced per molecule of acetic acid). Also, the thermodynamic efficiency of the FC was calculated on the basis of the converted ethanol and considering only ethanol oxidation to acetic acid, in consideration of the fact that acetic acid is the major product of ethanol oxidation in DEFCs [57]. The enthalpy change of ethanol oxidation to acetic acid is roughly one-third of the total enthalpy available from oxidation of ethanol to carbon dioxide. The rate of ethanol consumption in the FC was calculated from the coulombic data using our previous assumptions, although gravimetric analyses gave similar (but less accurate) results. This rate was multiplied by the enthalpy of oxidation of ethanol to acetic acid to obtain the chemical energy available to the FC. By dividing the calculated power output by the resulting enthalpy rate, we are able to obtain a power conversion efficiency for the FC.

### Cold trap

To verify the vapor composition (either before or after running it through a FC), a dry ice–ethanol cold trap (−80 °C) was used to condense any ethanol, water, acetic acid, and acetaldehyde in the vapor stream. Using FTIR and gravimetric analysis, we determined that the relative and absolute amounts of ethanol and water passing into the cold trap matched the values established from Wilson’s equation over the range of operating conditions used in this study (see Additional file 1: § Vapor–Liquid Equilibrium). These methods were also used to establish the product distribution in runs with FCs. In capturing volatile products from the FC effluent gas, amounts of acetic acid, acetaldehyde, water, and unused ethanol in the outlet stream were quantified.

## Results and discussion

### Vapor-fed direct ethanol FCs

Though liquid-fed operation of direct alcohol FCs is well-studied in the literature, little data exist for operation using a humidified ethanol vapor feed at near-ambient temperatures. Ghumman and Pickup used such a system to examine the effect of voltage pulses on carbon dioxide generation [57]. They used a low flow rate of 27 mL/min, a moderate ethanol concentration of 6% (1M), and flowing H_2_ at the cathode to maintain a stable reference. We can find no other studies of ambient-temperature ethanol vapor-fed FCs in the literature. We therefore first systematically studied the performance of this system, separate from any fermentation.

In vapor-fed FCs, the ethanol partial pressure should influence the FC performance in a manner similar to ethanol concentration in the liquid phase [58, 62, 65]. To examine the role of ethanol partial pressure, different gas mixtures were run through the FC anode at very high flow rates (1 L/min). Over the range of ethanol–water mixtures examined (0.5–15% ethanol), the partial pressure of water in the vapor feed remains roughly constant (within 5%) at 20.5 mmHg, while the 1 L/min flow rate ensures that the FC is consuming only a small fraction of the ethanol passing through (<5%). In this way, the formation of products and their effect on the reaction kinetics is small, and the concentrations of ethanol and water in the FC can be assumed to be constant.

**Fig. 2:**
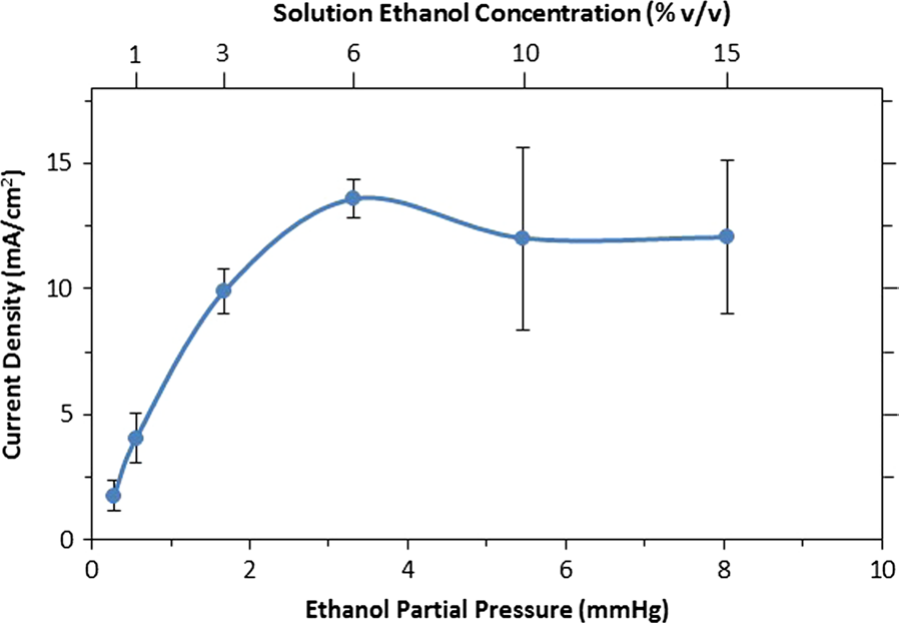
Current density versus ethanol partial pressure. Concentrations of ethanol in the bubbling solution are given at the top of the graph. The *line* is drawn to guide the eye. The *error bars* represent the standard deviation of three independent runs. As ethanol partial pressure increases, the current density increases, until a maximum is reached at 3.5 mmHg and 13.6 mA/cm^2^

Figure 2 shows the resulting current output of the FC, with a voltage fixed at 200 mV, as a function of ethanol partial pressure. A second *x*-axis at the top of the graph shows the solution ethanol concentration for reference. At low partial pressures, the current density increases nearly linearly with ethanol concentration, consistent with a pseudo-first-order reaction with respect to ethanol (rate constant 36 min^−1^). At higher ethanol partial pressures, the current density peaks near 3.5 mmHg at a value of 13.6 mA/cm^2^, slightly higher than the peak current density obtained when these same FCs are run on liquid ethanol–water mixtures at the same temperatures and voltage [39, 43, 48]. At higher ethanol partial pressures, there is a large run-to-run variability, along with an apparent slight decrease in current density. Similar decreases are observed in FCs running on liquid fuels [39, 67]. The decrease in performance likely arises from ethanol crossing over through the Nafion separator, which produces a mixed potential and lowers performance [36, 68]. Overall, these results demonstrate that FCs running on ethanol–water vapor can achieve power densities comparable to those for liquid-fed FCs, over a wide range of ethanol partial pressures.

**Fig. 3:**
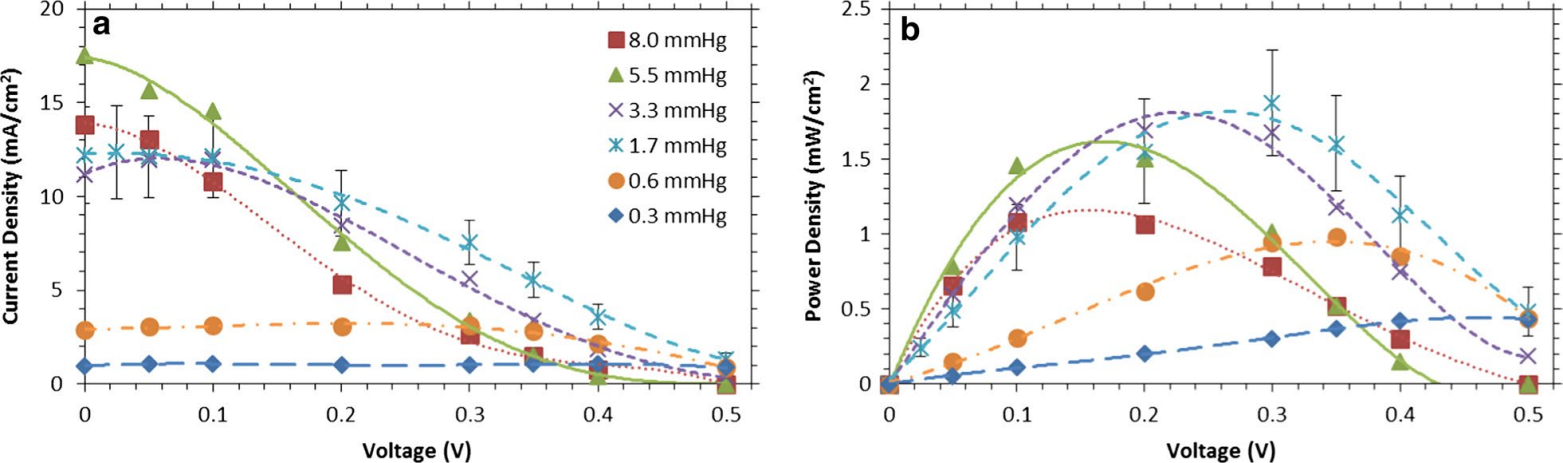
Current density (**a**) and power density (**b**) of the vapor-fed FC versus voltage, for a range of ethanol partial pressures. Three runs were done for the 1.7 mmHg ethanol data, and the standard deviation of the data is shown in the *error bars*. As the partial pressure increased, the peak power point shifted to lower voltages. Maximum power density of 2 mW/cm^2^ was obtained for 1.7 mmHg (3% ethanol in solution), at 280 mV. *Lines* are polynomial fits to guide the eye

To investigate the impact of ethanol partial pressure on performance at different operating voltages, the FCs were fed with the vapor from ethanol–water mixtures at a high flow rate (1 L/min) as shown in Fig. 2, but the potential was varied between 0 and 500 mV. The potential was stepped and held for 20 min to allow the FC to reach a steady state. The polarization curves and the calculated power curves for various ethanol concentrations are shown in Fig. 3.

As shown in Fig. 3a, as the ethanol concentration increases, current output reaches a maximum and then decreases, though the optimal concentration depends on voltage. At high potentials (near 500 mV), the current output shown in Fig. 3a peaks at a low ethanol partial pressure of 1.7 mmHg (3% ethanol in bubbling solution). Since the reaction is limited by charge transfer at high potentials, ethanol crossover is a significant impairment to current output, especially at higher ethanol concentrations. For example, at 5.5 and 8.0 mmHg (10 and 15% ethanol solutions, respectively), the crossover is significant enough that it has lowered the open-circuit potential to 400‒500 mV (as compared to 600‒700 mV at lower concentrations) resulting in zero net current. In contrast, near 0 mV, there is little resistance to charge transfer and the reaction is concentration or mass-transfer limited, so current increases with increasing ethanol partial pressure up to 5.5 mmHg. The small decrease in current output between 5.5 and 8.0 mmHg of ethanol (10 and 15% in solution) may be related to ethanol reducing H^+^ conductivity in the Nafion [69].

When considering the operating voltage at a particular concentration, the power density (Fig. 3b) is often more relevant than the current. As shown in Fig. 3a, the impact of ethanol crossover at high voltage is very apparent and causes a very large shift in the peak power towards lower voltages at higher ethanol partial pressures: 500–150 mV between 0.3 and 8.0 mmHg of ethanol. Interestingly, while peak power is low at both high and low ethanol concentrations, at intermediate ethanol partial pressures (i.e., between 1.7 and 5.5 mmHg) the peak power density changes by <25%, even though the optimal voltage shifts between 150 and 300 mV.

Similar effects have been noted in previous work with liquid-fed direct ethanol FCs, as well as work by others with direct methanol FCs. LSV data for a liquid-fed direct ethanol FC are shown in Additional file 1: Figure S5 to examine whether there are differences in optimal FC operating voltages between liquid-fed and vapor-fed. When comparing these identical FCs, the optimal voltages for a given ethanol solution are comparable up to 6% ethanol, though the instantaneous open-circuit voltages are slightly higher for vapor-fed direct ethanol FCs. The current and power densities, obtained at ambient temperature with an air-facing cathode, are broadly similar to other direct ethanol FCs, but it is likely that further enhancements could be obtained with additional engineering of the FCs, especially its cathode [70, 71]. The energy from ethanol oxidization to acetic acid is converted to electrical energy at efficiencies typical for direct ethanol and direct methanol FCs (approaching 40% at 500 mV) [25], but the overall process efficiencies are much lower, in large part due to the inability to fully oxidize ethanol to carbon dioxide. Additionally, only a small fraction of the ethanol is converted at high flow rates, although as discussed below higher single-pass conversions can be obtained by reducing the flow rate. Direct methanol FCs are known to suffer severe performance drops from methanol crossover, though systems that feed methanol as a vapor have been shown to mitigate crossover [72]. For both methanol and ethanol, vapor-fed operation may offer a means of reducing crossover, and control of the voltage allows for higher-power operation across a range of partial pressures.

**Fig. 4:**
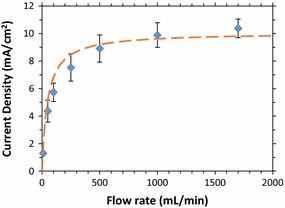
Current from vapor-fed FC fixed at 200 mV versus gas flow rate. Ethanol partial pressure was 1.7 mmHg, achieved by bubbling through 3% ethanol (*blue diamonds*). The *error bars* represent the standard deviation of three independent runs. The *red dashed line* is the prediction of the continuous stirred-tank reactor (CSTR) model, using the measured reactor volume and a rate constant obtained from the data shown in Fig. 2

The behavior of the FC versus flow rate was as expected from the current dependence on concentration (Fig. 2). We determined the dependence of current output on flow rate at constant potential (200 mV) and constant composition of the vapor stream (1.7 mmHg). Figure 4 shows how the current density varies with flow rate. An ethanol partial pressure of 3.1 mmHg falls in the pseudo-first-order region for ethanol partial pressure (see Fig. 2), allowing the current densities to be fit using a continuous stirred-tank reactor (CSTR) model with first-order reaction kinetics (for the equations used, see Additional file 1: § CSTR Model). The CSTR model was chosen because of the rapid mixing of gas expected in the fuel reservoir at the anode of the FC. Based on this model, and the rate constant determined based on the data shown in Fig. 2, a fit was obtained (red dashed line in Fig. 4) that is in excellent agreement with the flow rate current densities. At high flow rates (≥250 mL/min), the current densities were comparable to currents achieved with batch liquid-fed operation [39, 48, 63]. At low flow rates, the current drops substantially, since the effective ethanol concentration in the FC is lower due to conversion of the ethanol to acetic acid and other products.

**Fig. 5:**
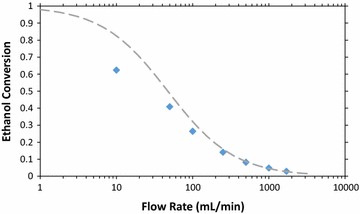
Ethanol conversion in the vapor-fed FC versus gas flow rate through 3% ethanol solution (ethanol partial pressure of 1.7 mmHg), with the FC fixed at 200 mV. Note the logarithmic *x*-axis. The *dashed green line* shows the CSTR prediction

The current data shown in Fig. 4 can also be used to determine an ethanol conversion that can be compared with results from gravimetric analyses. Figure 5 shows the coulombic conversion of ethanol in the vapor-fed FCs as a function of flow rate, for a fixed voltage (200 mV) and ethanol partial pressure (1.7 mmHg). The dashed line shows the expected conversion based on a CSTR model. Conversion to acetic acid (4 electrons per ethanol molecule) was used as the basis for the figure, because acetic acid is the primary product of ethanol oxidation [57], and because it represents a more complete oxidation of ethanol than conversion to acetaldehyde, the most common secondary product. Based on gravimetric analysis of the cold-trap condensate of the FC effluent gas, acetic acid is almost exclusively produced at high flow rates (~20:1 acetic acid-to-acetaldehyde ratio in products at 1000 mL/min). At lower flow rates the percent of acetaldehyde in the products increases, reaching 12% at 200 mL/min and 21% at 100 mL/min. In all cases, the mass balance based on the inlet vapor and condensate approximately closes (within 10%), as does the electron balance from the current output and condensate (assuming 2 electrons per molecule of acetaldehyde, 4 electrons per molecule of acetic acid, and no Faradaic losses). Ethanol conversions >60% are observed at our lowest tested flow rate of 10 mL/min.

**Fig. 6:**
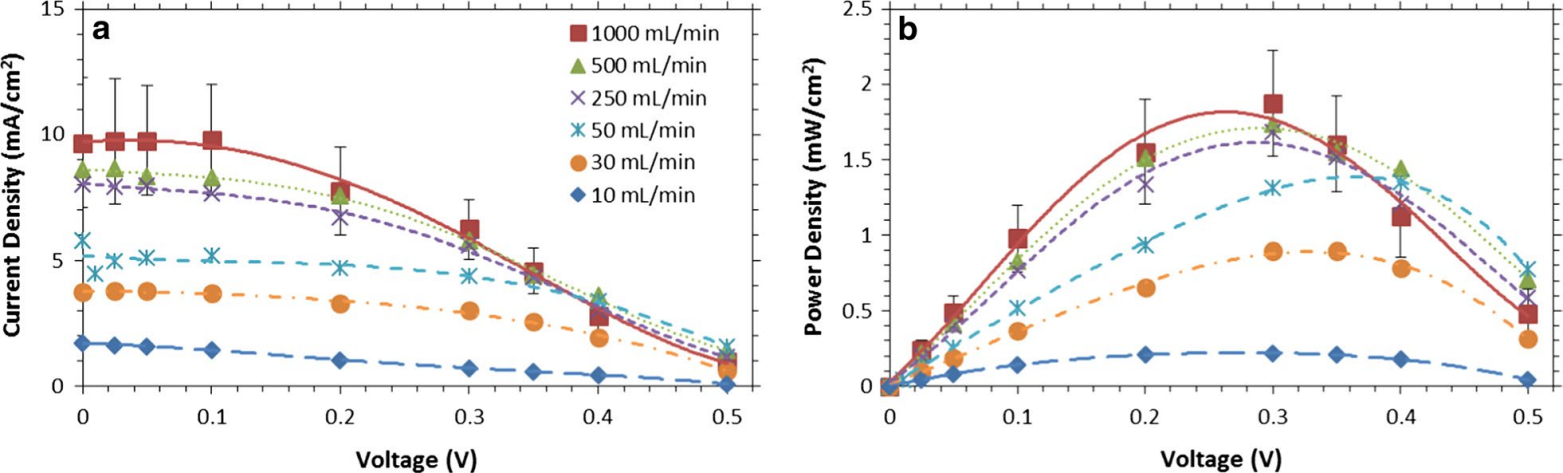
Current density (**a**) and power density (**b**) of the vapor-fed FC versus voltage, for a range of gas flow rates at 1.7 mmHg ethanol. Three runs were done for the 1000 mL/min ethanol data, and the standard deviation of the data is shown for the *error bars*. As the flow rate increased, the peak power increased, as expected. The peak power point also shifted to lower voltages. Maximum power density of 2 mW/cm^2^ was obtained for 1000 mL/min, at 280 mV. *Lines* are polynomial fits to guide the eye

The voltage response of the vapor-fed FC depends on the flow rate as shown in the variable voltage data plotted in Fig. 6. Figure 6a shows current as a function of voltage, while Fig. 6b shows power as a function of voltage. At very low flow rates, (<50 mL/min) the current and power increases for all voltages as flow rate increases. However, as the flow rate increases further, the current output increases only at low voltages (<400 mV). In this regime, the reaction is likely mass-transfer limited with charges created at the anode having little resistance flowing to the cathode [28]. In contrast, at higher voltages, the vapor-fed FC is likely limited by charge transfer between the anode and the cathode. These effects also shift the optimal voltage to higher voltages as the flow rate decreases. Though there is a 55% drop in current output at 200 mV when running the FC at 1000 mL/min as opposed to 50 mL/min, the peak power only drops 28% between 1000 and 50 mL/min. There is a trade-off between low flow rates that produce low power densities with high conversions and high flow rates that produce higher-power densities but may require the fuel to be recycled due to their low single-pass conversion. Since operating at lower flow rates may be desirable to reach higher conversions, the high power density available across a range of flow rates should be highly beneficial. Additionally, running the vapor-fed FCs at higher voltages should enable more of the ethanol’s energy to be captured as electrical power with minimal losses in the power density, especially at low flow rates.

### Bio-hybrid fuel cells

In “Vapor-fed direct ethanol FCs” section, we showed that vapor-fed FCs have similar performance to liquid-fed direct ethanol FCs when operating on ethanol–water mixtures. Here we show one of the major advantages of vapor-fed FCs: the ability to operate on complex mixtures such as fermentations that often contain components that poison FCs. To demonstrate these capabilities, the vapor-fed FC was run as a bio-hybrid FC with an ongoing yeast fermentation as the ethanol source. The fermentation was run with a rich medium (YPD) that is known to rapidly poison FCs [38]. Even with an RO separation membrane to help purify the ethanol from the fermentation, liquid-fed bio-hybrid FCs have a >90% reduction in power density over a period of 1 week when running on rich media [48].

Because ethanol was constantly being removed from the fermentation, the bubbled fermentation appeared to be maintainable indefinitely simply by adding sugar and water periodically. A nitrogen flow rate of 30 mL/min was used to ensure reasonable ethanol conversion by the FC; this flow rate was sufficient to keep the ethanol concentration of the 125 mL fermentation between 4 and 8% ethanol when 3.75 g of glucose was added twice per week (i.e., every 3–4 days). A preliminary 1-month bio-hybrid FC run at higher flow rates is shown in Additional file 1: Figure S5.

**Fig. 7:**
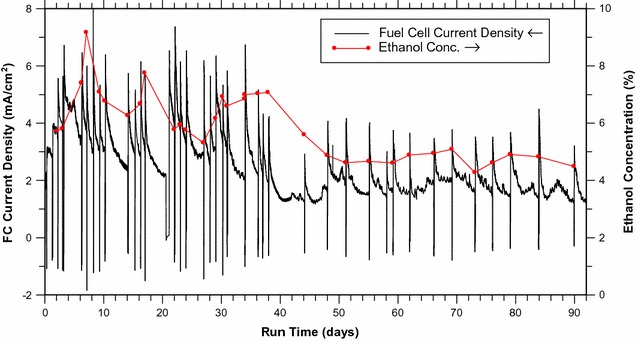
Long-term performance of a vapor-fed bio-hybrid FC versus run time, in days. Ethanol was stripped from a 125 mL yeast fermentation and fed into a FC (with area of 2.68 cm^2^). The FC current density (at 200 mV) was measured at intervals of 1 s, and is plotted as a *black line*, using the left-hand *y*-axis. The ethanol concentration of the fermentation was measured just prior to most feedings, and is plotted as *red dots*, using the right-hand *y*-axis (The *red line* is only a visual aid; it is not data.)

The vapor-fed FC can operate with an ongoing fermentation for at least 5 months while maintaining a power density >0.3 mW/cm^2^. The current density and fermentation ethanol concentration for the first 90 days are plotted in Fig. 7. The baseline vapor-fed FC characterization was used to provide an expected current density for the bio-hybrid FC based on the average ethanol concentration (4‒8%), the flow rate (30 mL/min), and the operating voltage (200 mV). Over the first 30 days, the ethanol concentration was roughly 7%, with fluctuations between 6 and 10%; the current density averaged 3.5 mA/cm^2^ while the expected current density for this ethanol concentration and flow rate was roughly 5 mA/cm^2^. This modest difference between the expected current density and the actual current density may be linked to the extended operation of the FC in the bio-hybrid FC configuration (months in Fig. 7 vs. hours in Figs. 2, 3, 4, 5). In month-long runs, current density can decline due to build up of reaction products on the catalysts and limit on the lifetime of the FC [73, 74]. Despite these issues, the FC runs nearly as well over the last 60 days, as shown in Fig. 7, as over the first 30 days, with a modest drop in current attributable to the decline in the fermentation ethanol concentration from roughly 7 to 4.5%. The hourly and daily average current densities for the entire 5-month run are shown in Additional file 1: Figure S6, § Averaged 5-Month Data. These results clearly demonstrate that the FC performance is maintained for at least 150 days when running as a bio-hybrid FC.

One interesting effect of the fermentation on the FC is that whenever sugar is added to the fermentation, spikes appear in the current density. These dramatic increases take several minutes to build up after reconnecting the gas flow. This time frame suggests the current density increases are caused by disrupting the gas flow and fermentation headspace. Purely electrical changes in the system would only require seconds to take effect. Increased ethanol in the fermentation would require hours, so that also cannot be the cause (although it may lengthen the effect). We can reproduce the effect by briefly substituting air for the ethanol/water vapor, but not by briefly cutting off the vapor flow, strongly suggesting oxygen as the cause. Addition of oxygen to the anode fuel stream induces a similar effect in hydrogen-fed proton-exchange membrane FCs, where it is thought to react with adsorbed carbon monoxide and reactivate the poisoned catalyst [75]. We therefore hypothesize that, after long-term operation of the FCs, oxygen clears catalytic sites of adsorbed CO species and/or other non-reactive molecules that are intermediates or products in ethanol electro-oxidation. Regardless of the reason, the increase in power lasts for days, which is 1000× longer than the several-minutes-long interruption. A practical device might do well to purposefully incorporate oxygen addition, either through inclusion of a small amount of air or through an alternating flow scheme.

**Fig. 8:**
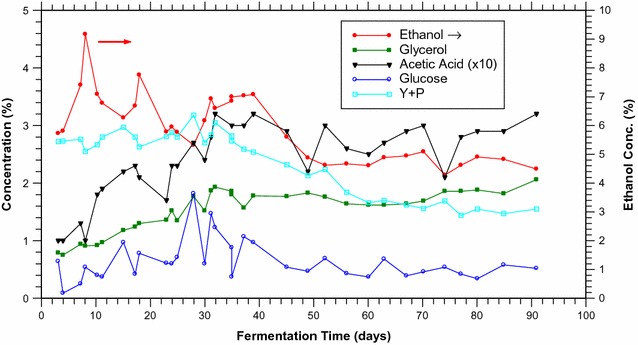
Major components of the bubbled fermentation versus time. *Y* + *P* = combined yeast extract and bacteriological peptone. Products are *closed symbols*; reactants are *open symbols*. Ethanol concentrations are plotted against the right-hand abscissa. The acetic acid concentrations actually range from 0.1 to 0.3%; they are plotted ×10. Connecting lines are intended only as visual aids for long-term trends; they are not data

The fermentation can also be run for very long times with little loss in ethanol production rate, allowing more ethanol to be produced than in a batch fermentation. Concentrations were obtained bi-weekly just prior to addition of glucose and water, via FTIR spectroscopy of small samples of the fermentation liquid, as explained in the “Fourier transform infrared (FTIR) spectroscopy” section. Since opening the fermentation causes current spikes as discussed above, samples were normally taken only when glucose and water were being added to the fermentation. Figure 8 shows the concentrations of the major components of the bubbled fermentation versus time, for about 90 days, while Additional file 1: Figure S7, § Control Fermentation shows the concentrations of major components of the control (non-bubbled) fermentation operating in the same conditions but without bubbling. In the control fermentation, ethanol builds up to 16% over 2 weeks, but then only another 2% is produced by the yeast. In contrast, when the fermentation is running with a bio-hybrid FC, the nitrogen bubbling prevents the ethanol concentration from building up beyond 10%, which would slow the fermentation and also be undesirable for FC operation. Eventually the ethanol concentration settles down to 4.5–5%, at the lower end of the concentration range for optimal FC operation. Since the rate of ethanol production in the control fermentation slows down noticeably once it has reached even modestly higher ethanol concentrations (ca. 10% and above), sugar accumulates in the fermentation. In contrast, because of the faster fermentation in the bubbled fermentation, the glucose concentration remains consistently low when samples are taken (normally <1%, although around day 25 some higher concentrations are observed). The low glucose concentrations indicate that the fermentation is sometimes glucose limited, and that more feeding would probably allow a higher ethanol concentration to be sustained, if desired. Even without optimizing the glucose addition rate, the yeast fermented 75 g of glucose over the course of 90 days. The gas leaving the fermentation, being saturated with ethanol, stripped 34 g of ethanol from the fermentation for use by the FC (45% of the glucose mass). Since the maximum theoretical yield is 39 g of ethanol (ignoring the yeast’s metabolic needs), the yield was 87%. By comparison, using the same strain and a fermentation in which a high initial concentration of glucose (30‒40%) is used, a yield of 66% is obtained (data not shown). Presumably the yeast grown for months with stripping produces higher ethanol yields than yeast grown for days in batch operation because the former are spending almost 100% of their time in stationary phase rather than growth phase. Similar yeast conditions can be obtained in batch operation, such as the technique used by Brazilian ethanol producers where yeast is collected and re-used at the end of a fermentation. This keeps the yeast in a metabolic state that produces ethanol, not biomass [15]. Yields of 91% are reported, comparable to our result of 87% [15].

The variations in the concentrations of the lesser components are also instructive. The concentrations of acetic acid (filled black triangles) and glycerol (filled green squares) both grow steadily for 30 days before plateauing and remaining stable for the next 60 days (note that the acetic acid concentration shown is ×10). Glycerol is commonly produced as a minor component by yeast, and builds up in the fermentation due to its low volatility. The acetic acid is probably also produced by yeast metabolism [76], rather than backflow from the FC, given its very low concentration in the fermentation. The low acetic acid concentration is crucial, since previous work has demonstrated that the amounts of acetic acid produced by a FC can wipe out a suitably sized fermentation within days if not excluded or suitably remediated [43]. Since the acetic acid is now a component of the output stream, it may be possible to break it down further in a separate reactor or microbial FC using bacteria that can consume acetate and produce fuel gases (e.g., methane), such as *Shewanella oneidensis* [77], *Anaeromxyobacter dehalogenans* [78], or *Geobacter sulfurreducens* [79]. Yeast extract and bacteriological peptone are low concentration components of the growth medium that provide yeast with material for cellular synthesis. Their combined concentration (*Y* + *P*, open cyan circles) is steady for about 25 days, and then gradually declines to about 3/5 of its original level. Concomitantly, a visible layer of dead yeast cells was observed at the bottom of the fermentation vessel, suggesting that some of these components were being trapped in dead cells. This decline in *Y* + *P* was slowing by 90 days suggesting that *Y* + *P* was being slowly released by deteriorating dead cells. Quick checks with light microscopy show many cells in the process of budding (data not shown). All of these observations indicate continued yeast cell formation, growth, and death, throughout the entirety of the 90 days.

## Conclusion

In this paper we have demonstrated the concept of an ambient-temperature (25 °C) vapor-fed bio-hybrid FC. We have shown that vapor-fed FCs have significant advantages for operating as bio-hybrid FCs with ongoing fermentations. Fermentations generally contain components that irreversibly poison a FC catalyst if placed in direct contact, but these components are normally non-volatile and therefore can be excluded when operating from a vapor feed. In the case of a yeast (*S. cerevisiae*) fermentation, the fermentation and the FC can operate together for at least 5 months with little decrease in the fermentation rate or FC current, in contrast to other bio-hybrid FC geometries where performance decreases markedly over 1 week [48].

We have also characterized the behavior of room temperature direct ethanol FCs fed with ethanol vapors and have shown that their performance is comparable to liquid-fed operation. As expected from results with methanol vapor-fed FCs, the ethanol vapor-fed FCs can operate near peak efficiency over a wide range of ethanol concentrations (2‒8 mmHg, equivalent to bubbling through 3‒15% ethanol solutions), in contrast to liquid-fed FCs which often have narrower operation regimes. Further enhancements in the power densities and power conversion efficiencies would be expected with additional engineering of the FC catalysts, membrane, and anode and cathode geometries. Given the long-term operation of the FC with the fermentation demonstrated here, it should now be possible to investigate these other areas without risk of damaging the FC. Because the FC and fermentation are not as intimately coupled as bio-hybrid FC designs based on separation membranes, it would also be possible to explore FC conditions (such as elevated temperatures) that are not biocompatible.

We also expect this bio-hybrid FC design to have benefits for the operation of fermenters. The ability to produce ethanol continuously is known to have a number of benefits in terms of ethanol production rates and overall ethanol yield; we have observed these benefits in our setup as well. Because the FC can run at reasonable power densities from vapors from dilute ethanol solutions, organisms can be selected for properties such as ethanol production rate or ability to digest complex substrates rather than maximum ethanol tolerance. In principle, the FC could also pull stripped gas from several organisms (which may be incompatible), in separate tanks with separate volumes and conditions, producing different fuels while degrading different components of a waste stream. For example, removing carbohydrates and ethanol at a first stage would force anaerobes at later stages to direct their slow but wide-ranging metabolism solely toward other components of the waste stream. These later stages would also take better advantage of the strengths of microbial FCs in degrading wastes and toxic chemicals [80–82], and in reducing total organic content (TOC) [81, 82], and could increase overall energy recovery. Finally, the active-vapor-fed bio-hybrid FC presented here relies on technology that is adaptable to a wide range of scales, from scavenging of smaller or remote biomass sources that would otherwise be neglected, to industrial-sized plants.

## Additional file

**Additional file 1.** Supporting Data and Analysis.

## Abbreviations

FC: fuel cell
RO: reverse osmosis
YPD: yeast peptone dextrose
IR: infrared
FTIR: Fourier transform infrared
HPLC: high performance liquid chromatography
PEM: proton-exchange membrane
MEA: membrane electrode assembly
LSV: linear sweep voltammogram
CSTR: continuous stirred-tank reactor
TOC: total organic content
