# Supplementary material for: Vapor-fed bio-hybrid fuel cell
Source: Biotechnol Biofuels. 2017 Mar 17;10:68. doi: 10.1186/s13068-017-0755-7 (PMC5356349; doi:10.1186/s13068-017-0755-7)
Supplement: Supplementary file 1 — Additional file 1. Supporting Data and Analysis. [file 13068_2017_755_MOESM1_ESM.pdf]

Supplemental Material for

Vapor-Fed Bio-Hybrid Fuel Cell

Marcus S. Benyamin †, Justin P. Jahnke †, and David M. Mackie †,*

Army Research Laboratory, 2800 Powder Mill Road, Adelphi, MD 20740, USA; mbenyami@terpmail.umd.edu (M.S.B.); justin.jahnke2.ctr@mail.mil (J.P.J.)

* Correspondence: david.m.mackie.civ@mail.mil; Tel.: +1-301-394-0925; Fax: +1-301-394-0310

† *These authors contributed equally to this work.*

Overall Setup

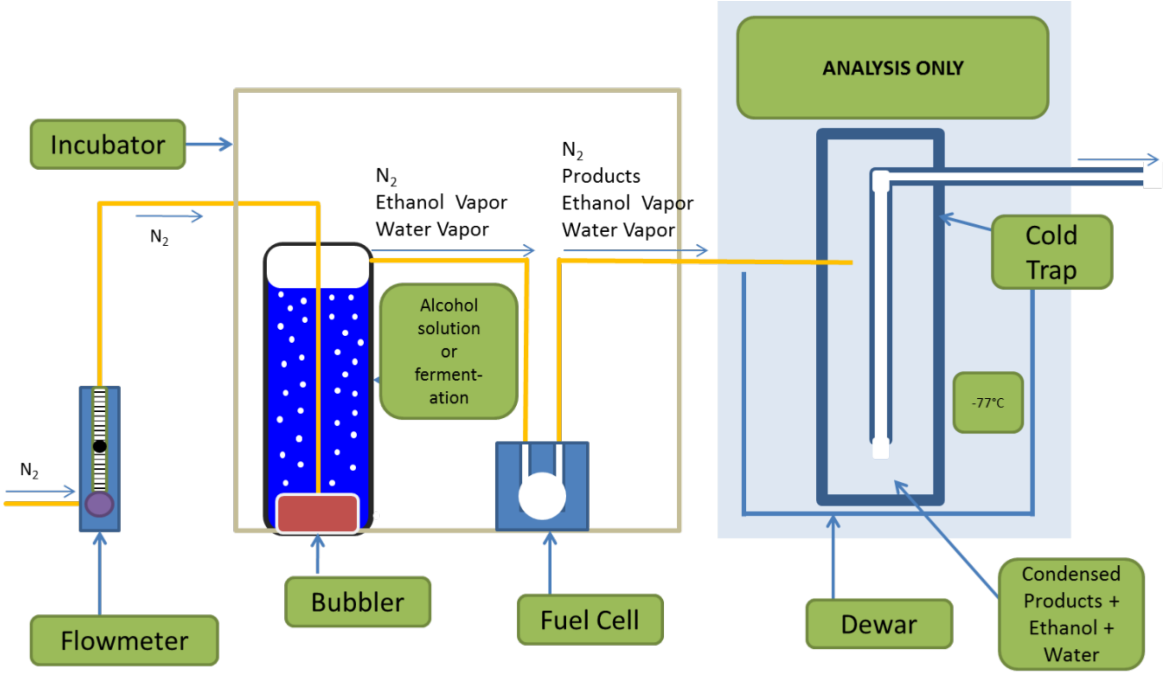

Figure S1. Schematic diagram of vapor-fed bio-hybrid FC as realized in a laboratory setting. (Compare to Figure 1.) A regulator (flowmeter) controls the flow rate of the sparging gas (here, N_2). An incubator is used to precisely control the temperature. A cold trap is optionally attached after the fuel cell (FC) to collect the FC products for gravimetric and FTIR analysis.

Vapor-Liquid Equilibrium

For ideal solutions, where all chemical components have similar properties, it is possible to determine the partial pressure exerted by a certain component in the vapor phase given the fraction of that component in the liquid phase. The relationship between the partial pressure and liquid composition is given by Raoult's Law:[1]

$$y_i P = P_i = x_i P_i^{sat} \quad \text{(S1)}$$

where y_i is the mole fraction of component i in the vapor phase, P is the total pressure, P_i is the partial pressure of component i, x_i is the fraction of i in the liquid phase, and P_i^{sat} is the vapor pressure of pure i at the given temperature.

In strongly nonideal systems such as ethanol-water mixtures, Raoult's Law fails to predict the partial pressure of the more dilute component (ethanol in the case of fermentation). The activity coefficient term γ_i corrects for solution nonideality, and in general, the relationship between the mole fraction of a volatile component in liquid solution and the partial pressure of that component in the vapor phase is given by a modified Raoult's Law:[1]

$$y_i P = P_i = \gamma_i x_i P_i^{sat} \quad \text{(S2)}$$

The activity coefficient depends strongly on both liquid-phase composition and temperature. Wilson's equation[1] is an empirical model that predicts the activity coefficients of each component and, applied to the ethanol-water system at room temperature, yields the curve shown in Figure S2, known as an XY diagram. Based on the predictions of Wilson's equation, it is possible to determine the partial pressures of ethanol and water at a given liquid composition and temperature. Further, since the flow rate of carrier gas is controlled in the experimental setup, it is possible to calculate the expected mass flow rates of ethanol and water vapor, which we confirmed using FTIR spectroscopy and gravimetric analysis. The data and standard deviations are shown in Figure S2.

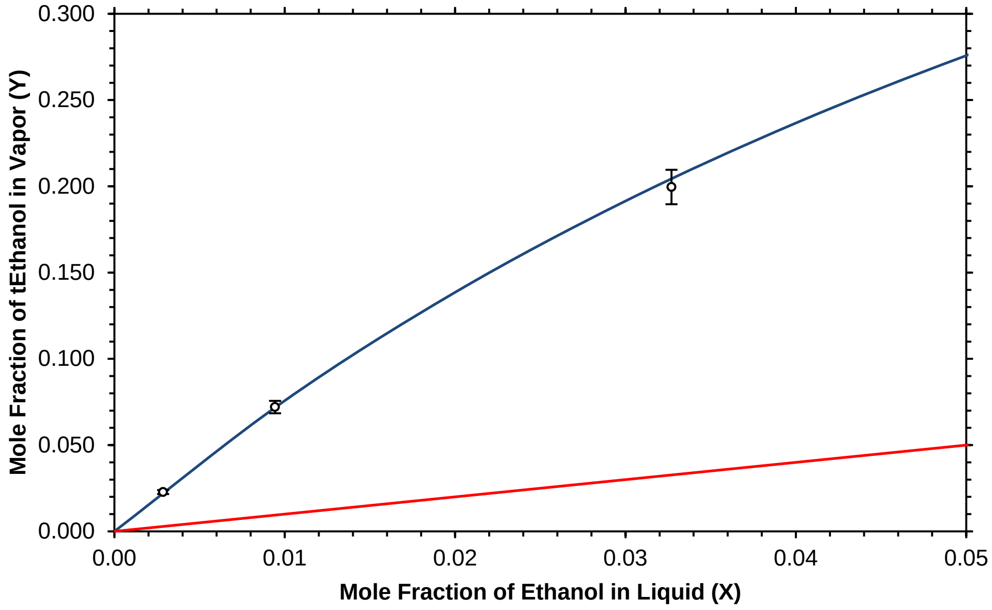

Figure S2. XY diagram for ethanol-water system at 25 C. The red curve is an X=Y reference line, and the blue curve represents the mole fraction in vapor of ethanol as calculated by Wilson's equation. Our data, collected to verify that the solution was indeed saturated and that the cold trap was trapping all of the volatile components, is shown as open black circles with standard deviation bars.

CSTR Model

The continuous stirred-tank reactor (CSTR) is a chemical reactor model in which there is a continuous inlet and outlet flow. In the ideal version of this model, the contents of the reactor are assumed to be well-mixed; that is, there are no temperature gradients or concentration gradients within the reactor, so the reaction occurs uniformly throughout the reaction volume. This also implies that the concentration of components in the outlet stream is identical to their concentrations in the reactor. This model is well suited to homogenous reactions in gaseous or liquid phase, and is useful in approximating some heterogeneous reactions that involve a solid phase.

From the diagram in Figure S3, we can write a mole balance for each chemical species. For chemical species A, at steady state,

$$N_{A,in} - N_{A,out} + N_{A,gen} = 0 \tag{S3}$$

where N indicates a molar rate. The left-hand terms are (left to right): inlet flow, outlet flow, and chemical generation (which will be negative if A is being destroyed). The right-hand side is the accumulation of A, which is zero at steady state.

For the direct ethanol FC, it is only necessary to consider the concentration of ethanol since the partial pressure of water remains nearly constant for each of the compositions tested.

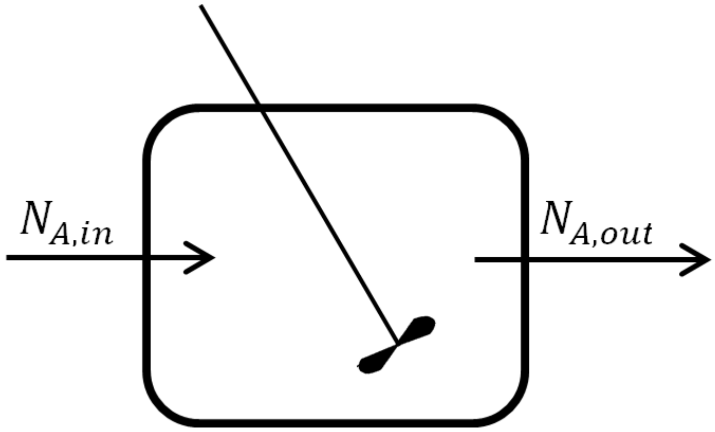

Figure S3. Diagram for continuous stirred-tank reactor, showing inlet and outlet streams.

In principle the generation term can have a complicated dependence on the concentrations of the various species in the reactor. However, Figure 2 shows that the reaction is pseudo-first order in ethanol partial pressure at low concentrations ($P_{EtOH} < 1.7$ mmHg). If we make this assumption, then the reaction rate (in mmHg/s) can be written as:

$$r = -kP_{EtOH,out} \tag{S4}$$

In the case of ethanol, we substitute the product of the reactor volume and the reaction rate for the generation term. Note that this new term must be converted to a molar rate using the ideal gas law.

$$N_{EtOH,in} - N_{EtOH,out} + \frac{V}{RT}r = 0 \tag{S5}$$

It is convenient to rewrite the molar flow rate terms (N_{EtOH}) in terms of the flow rates and partial pressures as follows:

$$N_{EtOH} = \frac{P_{EtOH}v}{RT} \tag{S6}$$

where P_{EtOH} is the partial pressure and v is the volumetric gas flow rate. By substituting equations S4 and S6 into S5 and rearranging, the outlet ethanol partial pressure can be obtained in terms of known quantities (V, v, k and $P_{EtOH,in}$):

$$P_{EtOH,out} = \frac{v}{v + kV} P_{EtOH,in} \tag{S7}$$

Using Faraday's constant with 4 electrons per molecule of ethanol oxidized, an equation for the fuel cell current density (j_{FC}) can also be obtained:

$$j_{FC} A_{FC} = \frac{4FV}{RT} r = \frac{4FV}{RT} k \frac{v}{v + kV} P_{EtOH,in} \tag{S8}$$

Equation S8 is plotted on Figure 4, using A_{FC} = 2.68 cm^2, k = 36 min^{-1}, V = 1.75 mL (the volume of the fuel reservoir) and $P_{EtOH,in}$ = 1.7 mmHg.

Finally, the conversion as a function of flow rate can also be obtained by substituting into its definition and rearranging:

$$X_A \stackrel{\text{def}}{=} 1 - \frac{N_{A,out}}{N_{A,in}} = \frac{kV}{v + kV} \tag{S9}$$

Equation S9 in plotted in Figure S4 using the same constants as in Figure 4.

Liquid-Fed Direct Ethanol Fuel Cells: LSV and Power Curves

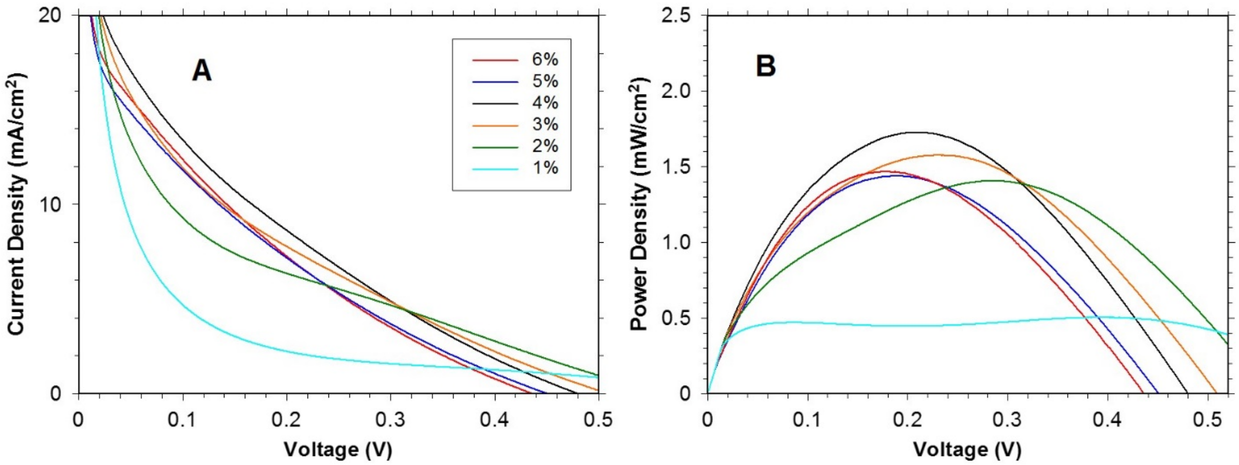

Figure S4. Current density (A) and power density (B) of a liquid-fed FC versus voltage, for a range of aqueous ethanol concentrations. Maximum power density of 1.73 mW/cm^2 was obtained for 4% ethanol, at 0.21 V. To ensure consistency, the FC was cleaned and run for 30 min before each data run. Lines are averages of at least three runs.

Preliminary Bio-hybrid Fuel Cell Experiment

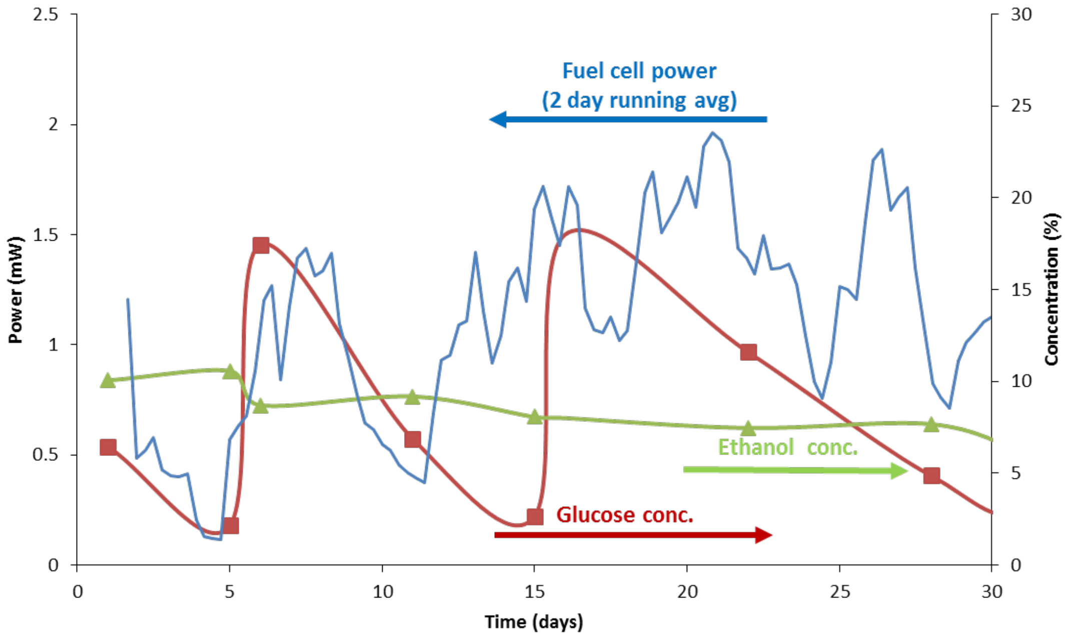

Figure S5: Preliminary run of vapor-fed fuel cell running on nitrogen gas bubbled through a fermentation. The fuel cell was held at a potential of 0.2 V and its power is shown on the left axis (MEA area = 2.68 cm^2). The glucose concentration (% wt/v)and ethanol concentration (% v/v) in the fermentation are shown on the right axis. Glucose was added on days 6 and 16. The fuel cell and fermentation were held at temperatures slightly above ambient (30°C) to be optimal for yeast fermentation. The fermentation volume was 250 mL and the flow rate was approximately 100 mL/min.

Averaged Five-Month Data

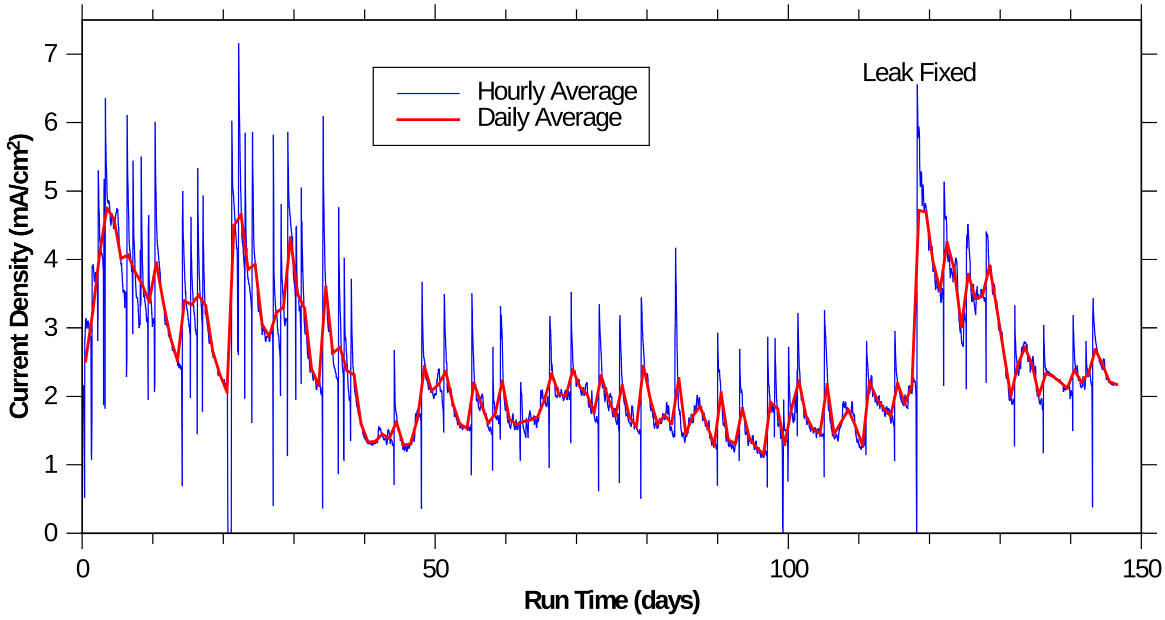

Figure S6. The long-term performance of a vapor-fed bio-hybrid FC is shown as a function of run time, in days. Ethanol was stripped from a 125 mL yeast fermentation

and fed into a FC (with area of 2.68 cm^2). The FC current density (at 200 mV) was measured at intervals of 1 s. The hourly and daily averages are plotted as blue and red lines, respectively. A leak in the vapor delivery was found and fixed on day 118.

Control Fermentation

Figure S7 shows the concentrations of the major components of the control (non-bubbled) fermentation vs. time, for about 70 days (rather than 90 days, as for the bubbled fermentation). As with Figure 7 for bubbling, the ethanol is plotted vs. the right-hand abscissa, and the acetic acid is x10. The control fermentation, as expected, quickly slows and stops due to yeast being unable to metabolize in high concentrations of ethanol. One obvious observation is that the glucose concentration is quite low in the 3, 7, 10, and 15 day samples. Together with the discussion of Figure 7, this again seems to indicate severe glucose limiting of the fermentation. The glycerol concentration initially plummets, then stays low (~0.5%) the rest of the time. The acetic acid concentration never exceeds 0.18%, and falls off over a long time (probably evaporating). These behaviors are different from the bubbling data. Between 23 days and 70 days, the glucose concentration doesn't increase, because we stopped feeding the non-bubbling fermentation after 23 days. (With no feeding, there was still 1.5% glucose at 70 days.) Lastly, the Y+P concentration is very steady, another notable difference from the bubbling fermentation, indicating limited yeast reproduction. We are following up to see if these apparent changes in the yeast's phenotype reflect changes in genotype.

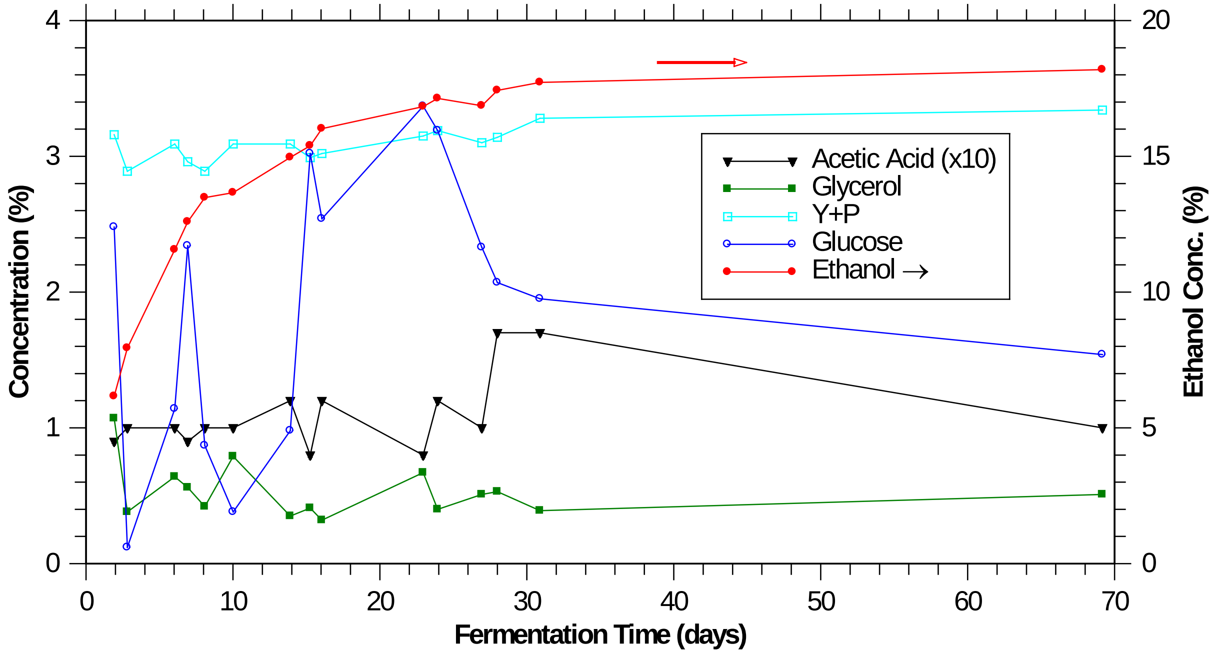

Figure S7. Major components of the control (non-bubbled) fermentation are plotted vs. time. Y+P = combined yeast extract and bacteriological peptone. Products are closed symbols; reactants are open symbols. Ethanol concentrations are plotted against the right-hand abscissa. The acetic acid concentrations actually range from 0.1% to 0.3%; they are plotted x10. The connecting lines are intended only as visual aids for long-term trends; they are not data.

----

1. J.D. Seader, Ernest J. Henley, D. Keith Roper, Separation Process Principles, 3rd Edition (2010) pp. 35–78, John Wiley & Sons
